# Exploring the Activation of the Keap1‐Nrf2‐ARE Pathway by PAHs in Children's Toys

**DOI:** 10.1111/cod.14792

**Published:** 2025-03-15

**Authors:** Jonas Lauenstein, Simon van de Weyer, Rasha Alsaleh, Christoph Wiedmer, Andrea Buettner, Christian Kersch, Simone Schmitz‐Spanke

**Affiliations:** ^1^ Institute and Outpatient Clinic of Occupational, Social, and Environmental Medicine Friedrich‐Alexander‐University of Erlangen‐Nuremberg Erlangen Germany; ^2^ Department 1—Agriculture, Ecotrophology and Landscape Development Anhalt University of Applied Sciences Bernburg Germany; ^3^ Chair of Aroma and Smell Research, Department of Chemistry and Pharmacy Friedrich‐Alexander‐Universität Erlangen‐Nürnberg Erlangen Germany; ^4^ Fraunhofer Institute for Process Engineering and Packaging IVV Freising Germany

**Keywords:** consumer goods, Keap1‐Nrf2‐ARE pathway, KeratinoSens assay, mixtures, polycyclic aromatic hydrocarbons, product safety, risk assessment, skin sensitization, synergistic effects

## Abstract

**Background:**

Children are particularly susceptible to environmental pollutants. This study assessed the skin sensitisation risk associated with polycyclic aromatic hydrocarbons (PAHs), prevalent in toys.

**Objectives:**

To evaluate the skin sensitisation potential of PAHs using the KeratinoSens assay.

**Methods:**

Individual PAHs (acenaphthylene, anthracene, benzo[a]anthracene, benzo[a]pyrene (B[a]P), benzo[b]fluoranthene (B[b]F), benzo[e]pyrene, benzo[g,h,i]perylene, benzo[k]fluoranthene (B[k]F), chrysene, fluoranthene, fluorene, naphthalene, phenanthrene, pyrene and triphenylene) and ternary mixtures containing B[a]P were assessed for their ability to activate the Keap1‐Nrf2‐ARE pathway in human keratinocytes. The concentration addition model and additive index were used to predict and analyse mixture effects.

**Results:**

Among the individual PAHs, B[k]F demonstrated the most potent activation of the pathway, exhibiting a 34‐fold higher potency relative to B[a]P. B[b]F, chrysene and B[a]P also exhibited significant activation, while the remaining PAHs displayed negligible or weak activation. Notably, PAH mixtures exhibited synergistic effects, except for those composed solely of potent sensitizers.

**Conclusions:**

This study provides the first assessment of the skin sensitization potential of these PAHs. The findings suggest that B[k]F, B[b]F and chrysene may pose a higher risk of skin sensitisation than previously thought. Additionally, the synergistic effects observed in mixtures highlight the importance of considering combined exposures when assessing PAH exposure risk.

## Graphical abstract

Our study evaluated the skin sensitisation risk of polycyclic aromatic hydrocarbons (PAHs) in toys, revealing that B[k]F, B[b]F and chrysene are highly potent sensitizers. Synergistic effects in PAH mixtures underscore the need to consider combined exposures when assessing environmental risks to children.

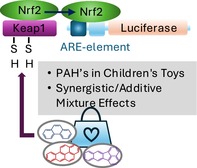

## 1 Introduction

Children are particularly vulnerable to the health risks of indoor air pollution due to their developing bodies, increased hand‐to‐mouth behaviour and higher breathing rates. Specifically, pollutants like semi‐volatile organic compounds, such as polycyclic aromatic hydrocarbons (PAHs), can pose health risks.

PAHs, a class of organic compounds with multiple aromatic rings, are ubiquitous environmental contaminants. Incomplete combustion of organic matter, such as fossil fuels, biomass and tobacco products, is a major source of PAHs. Indoors, sources include cooking with gas, burning wood or secondhand smoke.

Exposure to PAHs can occur through inhalation, ingestion and dermal contact. Skin contact can occur through direct contact with contaminated surfaces or through absorption. PAHs are recognised carcinogens, linked to an increased risk of lung, bladder and skin cancers. Additionally, exposure is associated with non‐cancerous health effects such as immune system suppression and respiratory tract inflammation [1].

Children are particularly susceptible to PAH exposure through indoor dust due to their hand‐to‐mouth behaviour and tendency to play close to the floor. Studies, such as one conducted in Canada that detected 12 PAHs in house dust samples from children's bedrooms (phenanthrene being the most prevalent), highlight this risk [2]. While smoking and attached garages are commonly associated with PAH exposure, children's toys can also be potential contributors. A collaborative research study investigated toys with offensive odours, isolating the volatile fraction and identifying the main odorants [3, 4, 5]. All samples were also subjected to a GC–MS screening for PAHs, which revealed the presence of up to 15 PAHs in a single toy sample (fancy dress accessory handbag) [4]. The rise in allergic contact dermatitis in children necessitates further research into potential risk factors. Atopic diseases are known to be associated with allergic sensitisation [6]. Some studies suggest a possible link between PAH exposure, particularly phenanthrene and pyrene, and an increased risk of asthma in children [7, 8]. However, the research on PAH sensitisation is limited and requires further investigation. While skin contact with low concentrations of naphthalene can cause severe dermatitis, the sensitising potential of other PAHs varies significantly. For instance, benzo[a]pyrene is considered a strong sensitiser, while (older) animal studies have not shown contact sensitisation for anthracene or phenanthrene [1, 9]. Critically, data on PAH skin sensitisation is currently lacking.

This study investigates the potential of PAHs identified in a children's handbag to activate the Keap1‐Nrf2‐ARE pathway. Specifically, the effects of 15 PAHs as individual substances and in ternary mixtures are examined: acenaphthylene, anthracene, benzo[a]anthracene (B[a]A), benzo[a]pyrene (B[a]P), benzo[b]fluoranthene (B[b]F), benzo[e]pyrene (B[e]P), benzo[g,h,i]perylene (B[g,h,i]P), benzo[k]fluoranthene (B[k]F), chrysene, fluoranthene, fluorene, naphthalene, phenanthrene, pyrene and triphenylene [4].

The Keap1–Nrf2–ARE pathway, a key cellular defence mechanism against electrophilic compounds and oxidative stress (known triggers of skin sensitization), regulates the expression of genes involved in antioxidant defence and detoxification. Under normal conditions, the transcription factor Nrf2 is bound to Keap1 and sequestered in the cytoplasm. When cells encounter electrophilic substances, these compounds covalently modify cysteine residues in Keap1, disrupting the Keap1–Nrf2 complex. This allows Nrf2 to translocate into the nucleus and bind to antioxidant response elements (AREs) in the promoter regions of various genes. These genes encode proteins involved in detoxification, antioxidant defence, and cell survival. Activation of this pathway, a key event in the skin sensitization adverse outcome pathway (AOP), can be assessed using the KeratinoSens assay [10]. This study's unique approach lies in examining the individual and combined effects of these PAHs on the Keap1–Nrf2–ARE pathway. By doing so, the study sheds light on potential interactions and synergistic effects between PAHs, which can inform future risk assessment strategies.

## 2 Methods and Materials

### 2.1 Chemicals

Chemicals were chosen and prepared following OECD test guideline 442D [10]. Cinnamic aldehyde (CAS no. 1437 1‐10‐9) and salicylic acid (99%, CAS no. 69‐72‐7) were purchased from Alfa Aesar (Thermofisher, Germany); ethylene glycol dimethacrylate (> 97.5%, CAS no. 97‐90‐5) was purchased from EMD Milipore Corporation (Germany); sodium dodecyl sulphate (SDS), (99%, CAS no. 151‐21‐3) from Sigma Aldrich (USA); and α‐pinene (CAS no. 80‐56‐8) from Acros Organics (USA). Test chemicals were dissolved in dimethyl sulfoxide (DMSO, CAS no. 67‐68‐5).

### 2.2 KeratinoSens Cell Line

Immortalised human keratinocytes (KeratinoSens cell line, Givaudan, Switzerland) were maintained in selection medium until 80% confluence. This medium consisted of Dulbecco's Modified Eagle Medium (DMEM, c.c.pro, Oberdorla, Germany) supplemented with 1 g/L glucose, 2% glutamine, 10% fetal bovine serum (FBS) and 500 μg/mL G418 (designated as KeratinoSens normal medium). For testing, cells were seeded at a density of 10,000 cells/well in 96‐well plates (clear for cytotoxicity, white for activation assays). Prior to testing, cells were cultured in G418‐free medium for 24 h containing 1% FBS at 37°C and 5% CO_2_.

### 2.3 KeratinoSens Assay (OECD Test Guideline 442D)

This study employed the KeratinoSens assay, validated by the European Union Reference Laboratory for Alternatives to Animal Testing (EURL ECVAM) following OECD Test Guideline 442D. KeratinoSens cells were exposed for 48 h to concentration ranges of single substances (0.5–1000 μM) and combinations (depending on cytotoxicity and luciferase induction) as detailed in the study design section [10, 11]. For luciferase activity, following incubation, cells were lysed using a commercially available passive lysis buffer (Promega, Germany). Luminescence was then measured with a modified luciferase substrate (20 mM tricine, 2.67 mM magnesium sulphate, 100 μM EDTA, 20 mM DTT, 125 μM ATP and 100 μM luciferin). Cell viability was assessed using the MTT (3‐(4, 5‐dimethylthiazolyl‐2)‐2, 5‐diphenyltetrazolium bromide) assay. After exposure, the medium was replaced with fresh medium containing 5 mg/mL MTT. Cells were incubated for 4 h at 37°C and 5% CO_2_. Following incubation, the supernatant was discarded, and formed crystals were dissolved with a solution of DMSO, acetic acid and SDS. After further incubation, absorbance was measured at 492 nm with a reference wavelength of 620 nm using a multimode microplate reader (Varioscan LUX 40, Life Technologies by Thermo Fisher Scientific, Waltham, USA).

The following endpoints were evaluated: Imax: maximal average fold induction of luciferase activity; EC1.5: concentration for which luciferase activity increased > 1.5‐fold compared to control; IC70: concentration at which a 30% reduction of cell viability occurs. A positive response was considered if the EC1.5 value was less than 1000 μM in at least two out of three repetitions, with a corresponding cellular viability exceeding 70%.

### 2.4 Study Design

The study employed a two‐part design. Individual PAHs were tested according to the OECD guideline in the first phase [10]. The second phase focused on testing mixtures, all of which included B[a]P due to its well‐established status as the most studied PAH. Seven ternary mixtures were investigated, with the ternary mixtures designed to group PAHs of similar structural characteristics. All the mixture experiments were conducted at fixed ratio concentrations, and chemicals were mixed according to prior testing (results not shown). The mixtures were diluted in 1:4 serial dilutions three times, and a full concentration response study was carried out. The individual chemical treatment was carried out in triplicate for each concentration, and two or three independent experiments were conducted for the mixture interaction studies. Details concerning the mixture compositions can be found in Table S1.

### 2.5 Prediction

Given that the mixtures comprised various PAHs and a similar mode of action (MoA) can be presumed, the concentration addition model (CA) was employed to predict the mixtures' activation of the Keap1–Nrf2–ARE pathway [12, 13, 14, 15].

(1) $$\mathrm{EC}1.5_{\mathrm{mix}}=\frac{1}{\sum_{i=1}^{n}\frac{p_{i}}{\mathrm{EC}1.5_{i}}}.$$

The concentration of the mixture predicted to induce a 1.5‐fold induction of the luciferase activity is denoted by EC1.5_mix_. EC1.5_i_ represents the individual correspondent concentration of component i within the mixture. Finally, *p*_i_ signifies the molar concentration ratio of the i‐th component relative to the entire mixture [14, 16]. The index of prediction quality (IPQ) is employed [16] to compare predictions obtained from the CA model with observed effects.

(2) $$\text{For}\;\mathrm{EC}1.5_{\text{predicted}}>\mathrm{EC}1.5_{\text{observed}},\;\mathrm{IPQ}=\left(\frac{\mathrm{EC}1.5_{\text{predicted}}}{\mathrm{EC}1.5_{\text{observed}}}\right)-1.$$

(3) $$\text{And}\;\mathrm{EC}1.5_{\text{predicted}}<\mathrm{EC}1.5_{\text{observed}},\;\mathrm{IPQ}=-\left(\frac{\mathrm{EC}1.5_{\text{observed}}}{\mathrm{EC}1.5_{\text{predicted}}}\right)+1.$$

An IPQ of zero indicates perfect prediction by the CA model. Negative IPQ values signify overestimation, while positive values indicate underestimation of mixture effects.

### 2.6 Determination of Interaction

The interaction of individual PAHs within the mixture was assessed using an additive index. This index uses the sum of the toxic activities (S) of the PAHs within their ternary mixtures as in Equation (4) [17, 18].

(4) $$S=\left(\frac{A_{m}}{A_{i}}\right)+\left(\frac{B_{m}}{B_{i}}\right)+\left(\frac{C_{m}}{C_{i}}\right).$$

*A*_*m*_‐*C*_*m*_ are the corresponding EC1.5 of the PAHs *A*‐*C* in the (ternary) mixtures, whereas *A*_*i*_‐*C*_*i*_ are the respective EC1.5 of the individual PAHs *A*‐*C*. The additive index was calculated based on the *S* values.

(5) $$\text{For}\;S\leq 1.0,\text{additive index}=\left(\frac{1}{S}\right)-1.0.$$

(6) $$\text{For}\;S\geq 1.0,\text{additive index}=S\left(-1\right)+1.0.$$

An additive interaction is indicated when the confidence interval spans 0. Conversely, a confidence interval > 0 suggests synergism, while an interval < 0 signifies antagonism.

### 2.7 Data Evaluation and Statistics

All experiments were performed at least two times in independent runs with three technical replicates each unless otherwise stated. Microplate reader readouts of fluorescence or absorbance measurements were normalised to control wells. Control values were set to 100% or 1 as needed.

## 3 Results

### 3.1 Individual PAHs

We investigated the activation of the Keap1‐Nrf2‐ARE pathway by 15 individual PAHs and compared the results to existing literature (Table 1). Because nearly no published data on the KeratinoSens assay, the local lymph node assay (LLNA), or the guinea pig maximisation test (GPMT) was available for the PAHs we tested (except for B[a]P), we used the H‐phrases (Health Hazards) of the Globally Harmonised System of Classification and Labelling of Chemicals (GHS) for further information [19].

**TABLE 1:** Comparison between literature data and study data for the studied PAHs.

		Literature data			Study data		
Substance	CAS	LLNA EC3%	GHS	Sensitization class	EC1.5 [μM]	IC 70 [μM]	Prediction [20]
Acy	208–96‐8	—	H302: Harmful if swallowed **H315: Causes skin irritation** **H319: Causes serious eye irritation** **H335: May cause respiratory irritation**	—	—	171	Nonsensitizer
An	120–12‐7	—	**H319: Causes serious eye irritation**	—	157.6	704	Sensitizer (weak)
B[a]A	56–55‐3	—	*H350: May cause cancer*	—	52.9	75	Sensitizer (moderate)
B[a]P	50–32‐8	0.0009[21]	**H317: May cause an allergic skin reaction** H340: May cause genetic defects *H350: May cause cancer* H360FD: May damage fertility or the unborn child	Extreme [21]	13.6	> 1000	Sensitizer (strong)
B[b]F	205–99‐2	—	*H35:0 May cause cancer*	—	0.5	> 1000	Sensitizer (strong)
B[e]P	192–97‐2	—	*H350: May cause cancer*	—	—	541	Nonsensitizer
B[g,h,i]P	191–24‐2	—	—	—	—	> 1000	Nonsensitizer
B[k]F	207–08‐9	—	*H350: May cause cancer*	—	0.4	903	Sensitizer (strong)
Chr	218–01‐9	—	H341: Suspected of causing genetic defects *H350: May cause cancer*	—	3.9	13	Sensitizer (strong)
Fla	206–44‐0	—	H302: Harmful if swallowed	—	—	85	Nonsensitizer
Fle	86–73‐7	—	—	—	177.9	> 1000	Sensitizer (weak)
Nap	91–20‐3	—	H302: Harmful if swallowed *H351: Suspected of causing cancer*	—	38.3	> 1000	Sensitizer (moderate)
Phe	85–01‐8	—	H302: Harmful if swallowed	—	83.4	> 1000	Sensitizer (moderate—weak)
Pyr	129–00‐0	—	—	—	—	118	Nonsensitizer
Trp	217–59‐4	—	**H318: Causes serious eye damage**	—	—	10	Nonsensitizer

*Note:* The relevant GSH classifications are highlighted in bold.

Abbreviations: Acy, acenaphthylene; An, anthracene; B[a]A, benzo[a]anthracene; B[a]P, benzo[a]pyrene; B[b]F, benzo[b]fluoranthene; B[e]P, benzo[e]pyrene; B[g,h,i]P, benzo[g,h,i]perylene; B[k]F, benzo[k]fluoranthene; Chr, chrysene; EC1.5, concentration for which the luciferase activity increased > 1.5‐fold; Fla., fluoranthene; Fle, fluorene; GHS, Globally Harmonised System of Classification and Labelling of Chemicals (only H‐phrases Health Hazards); LLNA, local lymph node assay; Nap, naphthalene; PAHs, polycyclic aromatic hydrocarbons; Phe, phenathrene; Pyr, pyrene; Trp, triphenylene.

Consistent with literature and H‐phrases, our study's findings on B[a]P's activation of the Keap1‐Nrf2‐ARE pathway are in line with the classification/prediction as a strong sensitiser with an EC1.5 of 13.6 μM. The lack of corresponding H‐phrases for the other PAHs is likely due to lacking data. Our findings reveal the activation of the Keap1‐Nrf2‐ARE pathway by the analysed PAHs in the following order, with B[k]F exhibiting the highest potential: B[k]F>B[b]F>chrysene>B[a]P>naphthalene>B[a]A> phenanthrene>anthracene>fluorene. The remaining PAHs acenaphthylene, B[e]P, B[g,h,i]P, fluoranthene, pyrene, and triphenylene did not activate the Keap1‐Nrf2‐ARE pathway in our study, suggesting a lack of sensitising potential. Among the investigated PAHs, chrysene and triphenylene exhibited the most potent cytotoxic effects as shown in Table 1. Figures S1 and S2 illustrate the dose–response curves of the individual PAHs for luciferase activation and cell viability.

### 3.2 Mixtures

Given the most extensive data availability for B[a]P, seven ternary mixtures encompassing all studied PAHs were designed, each containing B[a]P. These mixtures incorporated structural similarities between components (details in Table S1).

We investigated the effects of the mixtures on Keap1‐Nrf2‐ARE pathway activation. Table 2 provides the following parameters: observed EC1.5, predicted EC1.5 values derived from the CA model, IPQ values for prediction accuracy assessment, and the additive index to quantify the combined interactive effects of PAHs within the mixtures. Additionally, Figure S3 further illustrates the dose‐dependent luciferase induction and cytotoxicity, while Table S3 details the sum of the toxic activities of the studied PAHs mixtures.

**TABLE 2:** Prediction of the mixture effects and determination of mixture interactions.

	Mixture	EC1.5_observed_ [μM]	EC1.5_predicted_ [μM]	IPQ	Additive Index	Interaction
1	B[a]P + Fle + Nap	10.1	85.2	7.4	9.7	Synergistic
2	B[a]P + An + Phe	6.1	85.8	13.1	13.7	Synergistic
3	B[a]P + B[b]F + B[k]F	0.7	0.6	−0.1	−0.2	Additive
4	B[a]P + B[a]A + Chr	1.2	18.3	14.7	17.1	Synergistic
5	B[a]P + B[e]P + B[g,h,i]P	5.9	378.6	63.2	63.9	Synergistic
6	B[a]P + Acy + Fla	3.1	200.4	62.8	63.9	Synergistic
7	B[a]P + Trp + Pyr	1.8	92.7	49.5	49.5	Synergistic

*Note:* The colour coding visually differentiates the irritant and sensitising potential of the investigated PAHs: irritants (An, Acy); severe eye damaging (Trp); sensitizer—weak (An, Fle), moderate (B[a]A, Nap, Phe), strong (B[a]P, B[b]F, B[k]F, Chr); nonsensitizer (Acy, B[e]P, B[g,h,i]P, Fla., Pyr).

Abbreviations: Acy, acenaphthylene; An, anthracene; B[a]A, benzo[a]anthracene; B[a]P, benzo[a]pyrene; B[b]F, benzo[b]fluoranthene; B[e]P, benzo[e]pyrene; B[g,h,i]P, benzo[g,h,i]perylene; B[k]F, benzo[k]fluoranthene; Chr, chrysene; Fla., fluoranthene; Fle, fluorene; Nap, naphthalene; Phe, phenanthrene; Pyr, pyrene; Trp, triphenylene; EC1.5, concentration for which the luciferase activity increased > 1.5‐fold; IPQ, index on prediction quality; IPQ value < 0 indicates overestimation; value > 0 indicates underestimation; value ≈ 0 indicates perfect prediction; additive index ≈ 0 indicates additive interaction, < 0 indicates antagonistic interaction, > 0 indicates synergistic interaction.

The individual EC1.5 values of the tested PAHs were found to be lower in all mixtures compared to their respective values after single exposure (Table S3). For instance, the EC1.5 value of B[a]P ranged from 0.2 to 0.3 μM in the mixtures, while it was 13.6 μM after single exposure. Notably, PAHs that did not exhibit any effect on the pathway after single exposure, such as acenaphthylene, B[e]P, B[g,h,i]P, fluoranthene, pyrene and triphenylene, were observed to activate the pathway in the mixtures. Assuming a comparable MoA due to the exclusive composition of PAHs in the mixtures, we employed the CA model to predict their effect on pathway activation (Table 2). Intriguingly, the observed EC1.5 values for all mixtures were lower than the predicted EC1.5 values, indicating an underestimation of the real effect. The sole exception was the mixture composed solely of strong sensitizers (B[a]P, B[b]F, B[k]F), where the observed effect was accurately predicted by the CA model.

To further investigate the interactions between PAHs in the mixtures, we employed the additive index approach (Table 2). The additive index values for all mixtures (> 0) indicate a synergistic effect, suggesting that the combined effect of the PAHs is greater than the sum of their individual effects. Once again, the mixture composed solely of strong sensitizers (BaP, B[b]F, B[k]F) stood as an exception, exhibiting an additive index value (≈ 0) consistent with an additive effect. The effects of the mixtures on cell viability are given in Table S2, showing the IC70 values of the individual PAHs within the mixtures being lower than the values after single exposure.

## 4 Discussion

This study explores the potential health risks posed by PAHs, a group of organic compounds found in indoor environments, particularly those where children play. While common sources of PAH exposure are well known, this study highlights toys as an overlooked potential source. The PAHs investigated in this study were all identified in a children's toy handbag at relevant concentration levels [4]. Given the increasing prevalence of allergic contact dermatitis, PAHs warrant investigation as potential triggers, especially considering their demonstrated ability to penetrate human and animal skin [1, 22].

The study examined the potential of individual PAHs and ternary mixtures, all containing B[a]P, to activate the Keap1‐Nrf2‐ARE pathway. The mixtures were designed to include PAHs of similar structure. This design mirrors real‐life situations where the named substances rarely occur singly but commonly as complex mixtures at relevant levels. We further focused on the Keap1‐Nrf2‐ARE pathway because it represents a key event in the AOP for skin sensitisation. This pathway can be efficiently evaluated using the KeratinoSens assay, which adheres to OECD guidelines [10]. It is important to acknowledge, however, that our study only investigated this single event within the complex pathway leading to skin sensitisation. To the best of our knowledge, this study represents the first attempt to assess the skin sensitisation potential of the majority of these compounds. Limited data suggest a strong sensitising potential for B[a]P [1, 9]. Alalaiwe et al. conducted a comprehensive study investigating the potential of six PAHs—naphthalene, fluoranthene, pyrene, chrysene, 1,2‐benzanthracene and B[a]P—to induce skin inflammation. Their analysis encompassed cytotoxicity, cyclooxygenase (COX)‐2, prostaglandin E2 (PGE2), chemokines and differentiation proteins [22]. Muthusamy et al. examined the effects of multi‐component mixtures of PAHs (B[a]P, naphthalene, phenanthrene, and pyrene) and heavy metal/loid(s) on the Keap1‐Nrf2‐ARE pathway in HepG2 cells [14].

Two primary mechanisms have been proposed to explain the effects of PAHs on Nrf2 signalling [23]. The most widely accepted hypothesis posits that PAHs induce oxidative stress, leading to increased levels of reactive oxygen species (ROS) and electrophilic metabolites. These reactive species can directly modify cellular components, disrupt redox homeostasis, and ultimately activate Nrf2 [24].

PAHs are frequently metabolised by cytochrome P450 enzymes, leading to the formation of both electrophilic metabolites and ROS. While this study did not quantify ROS generation following PAH exposure, a limited number of studies have investigated this aspect. In liver cells, B[a]A induced higher ROS levels compared to B[k]F, and phenanthrene induced higher levels compared to B[b]F [25, 26]. Interestingly, in our study, B[a]A and phenanthrene were predicted as moderate sensitizers, whereas B[k]F and B[b]F were predicted as stronger sensitizers, which appears to contradict this hypothesis.

Cellular redox homeostasis is significantly influenced by the antioxidant capacity, particularly the levels of glutathione. However, there are few studies comparing glutathione levels after exposure to different PAHs. In contrast to the previously mentioned higher ROS levels in liver cells after phenanthrene exposure compared to B[b]F exposure, total glutathione content was higher after B[b]F exposure than after phenanthrene exposure in the same study [26]. No differences were observed in the activity of glutathione‐related enzymes. Nevertheless, this study demonstrated significantly increased Nrf2 levels in the nuclear fraction following B[b]F exposure compared to phenanthrene exposure, consistent with our prediction. Previous studies have reported conflicting findings regarding the effects of B[a]P and its metabolites on Nrf2 activation. Souza et al. demonstrated the activation of Nrf2 by B[a]P and its 9,10‐diol metabolite in liver cells, whereas Nguyen et al. reported that B[a]P, but not its metabolites, increased nuclear Nrf2 protein levels in Jurkat cells through downregulation of Keap1 [27, 28].

A second proposed hypothesis involves a functional interaction between the aryl hydrocarbon receptor (AhR) and the Nrf2 transcription factor [23]. PAHs, acting as ligands for the AhR, induce its activation, leading to the upregulation of phase I drug‐metabolising enzymes, such as cytochromes P450. In mouse liver cells, PAH‐induced Nrf2 activation has been shown to be dependent on AhR, as siRNA‐mediated knockdown of AhR abolished this effect, indicating a direct link between these two signalling pathways [29]. The extent of AhR activation by different PAHs varied considerably. Goedtke et al. demonstrated that B[k]F was the most potent AhR activator among the PAHs tested, followed by B[b]F, B[a]P, and B[a]A [30]. These findings correlate with our prediction of B[k]F, B[b]F, and B[a]P as strong sensitizers. In line with this, fluoranthene, phenanthrene and pyrene were shown to be either weak or non‐AhR activators, consistent with their classification as non‐sensitizers or weak sensitizers in our study.

These findings suggest a complex relationship between ROS levels and sensitisation potential, highlighting the need for further research to fully elucidate the underlying mechanisms. Furthermore, our results provide strong evidence for the involvement of AhR in Nrf2 activation, with potent AhR activators exhibiting stronger sensitisation potential.

Across all mixtures, all PAHs activated the Keap1‐Nrf2‐ARE pathway at significantly lower concentrations. Even the PAHs that showed no effect in single‐compound exposures led to activation in their mixtures. We employed the concentration addition (CA) model to predict mixture toxicity [12, 15, 31]. As previously mentioned, AhR activation is a shared molecular mechanism underlying PAH action, prompting us to adopt the concept of addition [32]. However, the model underestimated the combined effects, as evidenced by the significant difference between predicted and observed EC1.5 values. This underestimation varied across mixtures, as further supported by the IPQ values. Interestingly, the model accurately predicted the combined toxicity of the mixture containing B[a]P, B[b]F and B[k]F, all predicted strong sensitizers individually. This suggests an additive interaction for strong sensitizers, contrasting with the synergistic effects observed in other mixtures. To confirm this, we employed the additive index approach, which revealed consistent synergism in all other mixtures except the strong sensitizer mixture, which again exhibited additivity.

Bloch et al. vividly described the complexities of mixture toxicity, likening additive effects to a “multi‐headed dragon” and synergistic effects to the “synergy of evil” [32]. This conceptualization implies that synergistic interactions often involve an enhancer mechanism driven by one or more components. Our findings suggest that PAH mixtures may influence CYP activity, potentially explaining observed effects. While compounds like fluoranthene, phenanthrene and pyrene modestly increased CYP1A2 activity, B[a]P, B[b]F and B[k]F significantly upregulated it [30]. This observation hints at a potential mechanism where weak and moderate sensitizers enhance the metabolic activation of potent compounds like B[a]P within mixtures. However, in mixtures containing strong sensitizers, this effect might be less pronounced, possibly due to CYP enzyme saturation. Further research is necessary to fully understand the underlying mechanisms of these interactions.

In conclusion, this study demonstrates the potential health risk posed by PAHs, particularly those found in children's toys, through their ability to activate the Keap1‐Nrf2‐ARE pathway, a key event in skin sensitization. Our findings highlight the importance of considering mixture effects, as synergistic interactions can significantly amplify PAH toxicity. The differential activation of the AhR receptor by PAHs appears crucial in determining their skin sensitization potential. While this study provides novel insights into PAH‐induced skin sensitization, it is limited by its focus solely on the Keap1‐Nrf2‐ARE pathway and the investigation of ternary mixtures. Additionally, the generalizability of findings to more complex mixtures requires further exploration. Notably, our results indicate that a simple additive model consistently underestimated the combined toxicity of PAH mixtures. Nevertheless, the study's strengths lie in its use of a well‐established assay and the identification of B[k]F as a potent sensitizer, providing a valuable contribution to the field of PAH toxicology. These findings are further important in the process of transforming current production practices into a more sustainable and health‐oriented scheme, and an urgently needed step in raising awareness for products of daily use, especially considering the young—for a sustainable and safe future.

## Author Contributions

**Jonas Lauenstein:** methodology, investigation, writing – review and editing, data curation, conceptualization, formal analysis, visualization. **Simon van de Weyer:** conceptualization, methodology. **Rasha Alsaleh:** writing – review and editing. **Christoph Wiedmer:** conceptualization, investigation, methodology, validation. **Andrea Buettner:** conceptualization, investigation, methodology, validation. **Christian Kersch:** formal analysis, software, methodology, conceptualization, investigation, data curation, visualization. **Simone Schmitz‐Spanke:** conceptualization, investigation, funding acquisition, writing – original draft, writing – review and editing, methodology, project administration, supervision, resources.

## Conflicts of Interest

The authors declare no conflicts of interest.

## Supporting information


**Data S1.** Supporting Information.

## Data Availability

The data that support the findings of this study are available from the corresponding author upon reasonable request.
