# Supplementary material for: Exploring the Activation of the Keap1‐Nrf2‐ARE Pathway by PAHs in Children's Toys
Source: Contact Dermatitis. 2025 Mar 15;93(1):31–8. doi: 10.1111/cod.14792 (PMC12134455; doi:10.1111/cod.14792)
Supplement: Supplementary file 1 — Data S1. Supporting Information. [file COD-93-31-s001.docx]

**Supporting Information**

**Exploring the Activation of the Keap1-Nrf2-ARE Pathway by PAHs in Children's Toys**

Jonas Lauenstein^a^, Simon van de Weyer^a^, Rasha Alsaleh^a^, Christoph Wiedmer^b^, Andrea Buettner^c,d^, Christian Kersch^a^, Simone Schmitz-Spanke^a*^

^a^Institute and Outpatient Clinic of Occupational, Social, and Environmental Medicine, Friedrich-Alexander-University of Erlangen-Nuremberg, Henkestr. 9–11, 91054 Erlangen, Germany

[jonas.lauenstein@uk-erlangen.de; simon@vandeweyer.de](rewritten://a330ec0d-fa20-4dde-98b5-4e1e084ef1f0); rasha.alsaleh@fau.de; christian.kersch@fau.de; simone.schmitz-spanke@fau.de

^b^Department 1 – Agriculture, Ecotrophology and Landscape Development, Anhalt University of Applied Sciences, Strenzfelder Allee 28, 06406 Bernburg, Germany

[christoph.wiedmer@hs-anhalt.de](mailto:christoph.wiedmer@hs-anhalt.de)

^c^Chair of Aroma and Smell Research, Department of Chemistry and Pharmacy, Friedrich-Alexander-Universität Erlangen-Nürnberg, Henkestr. 9, 91054, Erlangen, Germany

^d^Fraunhofer Institute for Process Engineering and Packaging IVV, Giggenhauser Str. 35, 85354, Freising, Germany.

[andrea.buettner@fau.de](mailto:andrea.buettner@fau.de)

*Corresponding Author:

Simone Schmitz-Spanke

simone.schmitz-spanke@fau.de

Institute and Outpatient Clinic of Occupational, Social, and Environmental Medicine, University of Erlangen-Nuremberg, Henkestr. 9-11, 91054 Erlangen, Germany

Phone: +49 09131/85-22255; Fax: +49-9131-8526102 (Simone Schmitz-Spanke)

ORCID: 0000-0002-0416-8236

The authors have no conflicts of interest.

**Table of Contents:**

Supporting Material and Methods

Supporting Results

**Supporting Material and Methods**

**Table S1**: Concentrations of individual PAHs in the multi-component mixtures for activation of the Keap1-Nrf2-ARE pathway in KeratinoSens cells.

|  | **Mixtures** | **Acy [µM]** | **An [µM]** | **B[a]A [µM]** | **B[a]P [µM]** | **B[b]F [µM]** | **B[e]P [µM]** | **B[g,h,i]P [µM]** | **B[k]F [µM]** | **Chr [µM]** | **Fla [µM]** | **Fle [µM]** | **Nap [µM]** | **Phe [µM]** | **Pyr [µM]** | **Trp [µM]** |
| --- | --- | --- | --- | --- | --- | --- | --- | --- | --- | --- | --- | --- | --- | --- | --- | --- |
| 1 | B[a]P + Fle + Nap |  |  |  | 13.63 |  |  |  |  |  |  | 333 | 83 |  |  |  |
| 2 | B[a]P + An + Phe |  | 167 |  | 13.63 |  |  |  |  |  |  |  |  | 167 |  |  |
| 3 | B[a]P + B[b]F + B[k]F |  |  |  | 13.63 | 20 |  |  | 10 |  |  |  |  |  |  |  |
| 4 | B[a]P + B[a]A + Chry |  |  | 52.91 | 13.63 |  |  |  |  | 7.81 |  |  |  |  |  |  |
| 5 | B[a]P + B[e]P + B[g,h,i]P |  |  |  | 13.63 |  | 31.25 | 333 |  |  |  |  |  |  |  |  |
| 6 | B[a]P + Acy + Fla | 125 |  |  | 13.63 |  |  |  |  |  | 62.5 |  |  |  |  |  |
| 7 | B[a]P + Trp + Pyr |  |  |  | 13.63 |  |  |  |  |  |  |  |  |  | 62.5 | 16.5 |

Chemicals were mixed in a fixed concentration ratio based on their individual ECIR1.5 value or cytotoxicity and diluted in 1: 4 serial dilution three times and a dose response study was carried out. The chemical mixtures and vehicle control were treated in triplicate for each experiment. Three technical replicates were used for all each mixtures in at least two independent experiments.

The color coding visually differentiates the irritant and sensitizing potential of the investigated PAH: irritants (An, Acy); severe eye damaging (Trp); sensitizer - weak (An, Fle), moderate (B[a]A, Nap, Phe), strong (B[a]P, B[b ]F, B[k]F); nonsensitizer (Acy, B[e]P, B[g,h,i]P, Fla, Pyr)

Abbreviations: Acy, Acenaphthylene; An, anthracene; B[a]A, benzo[a]anthracene; B[a]P, benzo[a]pyrene; B[b]F, benzo[b]fluoranthene, benzo[e]pyrene, B[e]P; B[g,h,i]P, benzo[g,h,i]perylene; B[k]F, benzo[k]fluoranthene; Chr, chrysene; Fla, fluoranthene; Fle, fluorene; Nap, naphthalene; Phe, phenanthrene; Pyr, pyrene; Trp, triphenylene

**2. Supporting Results**

**
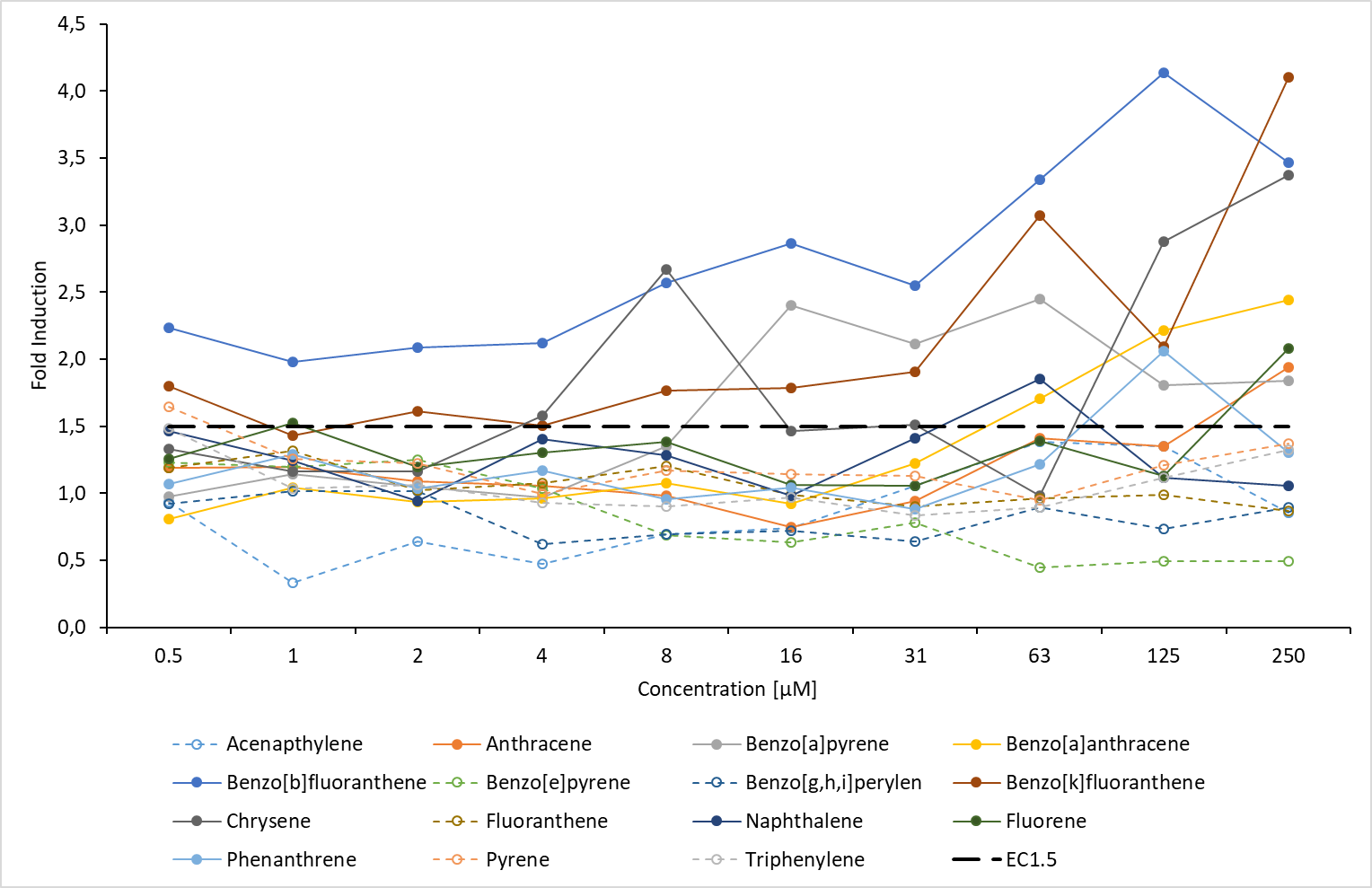
2.1. Individual PAHS**

**
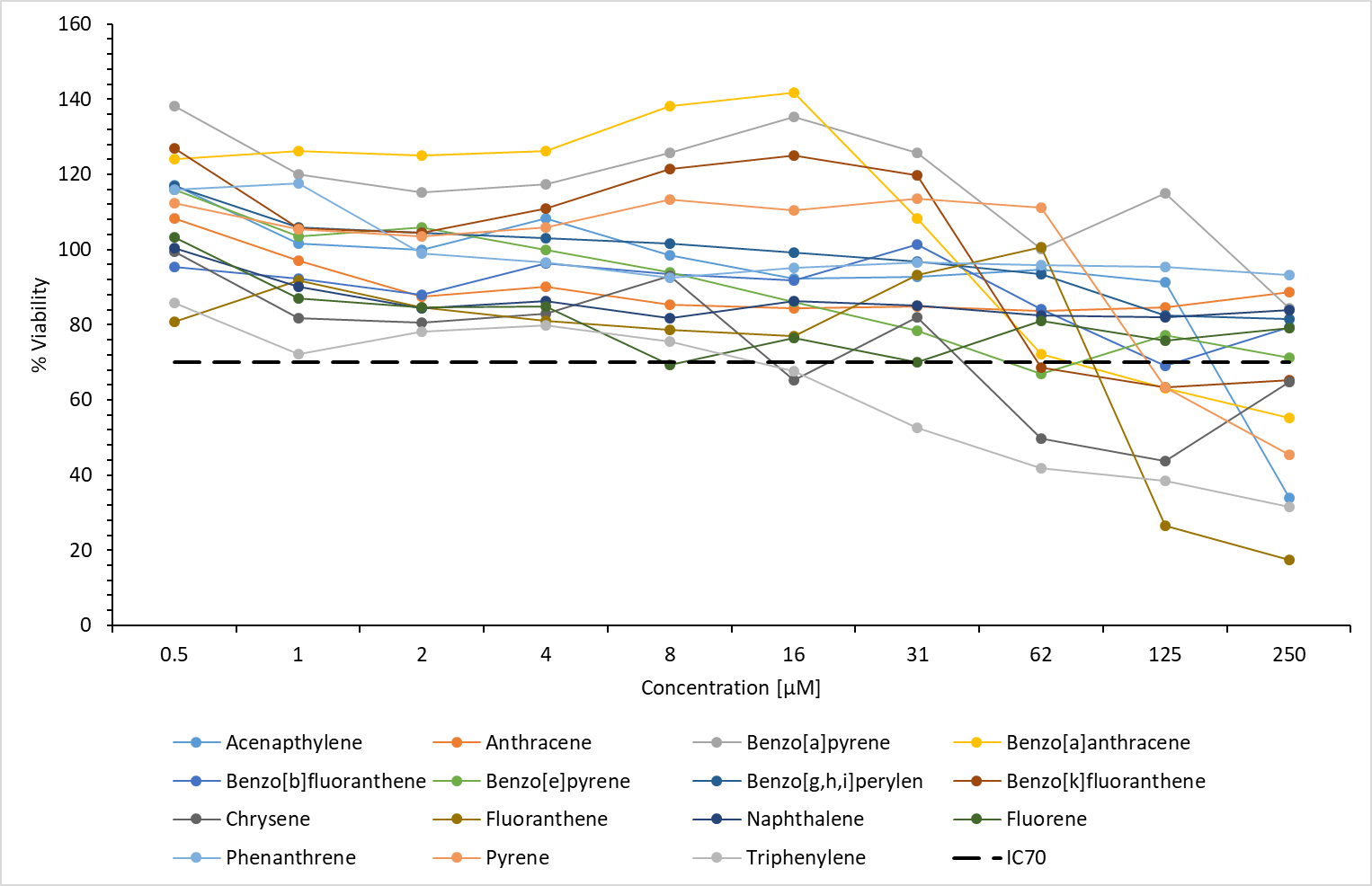
Figure S1** shows the induction values of the luciferase activities of the individual PAHs. The dashed line represents the EC1.5 criterion of the OECD guideline. Open circles represent the luciferase activity of PAHs that are not predicted to be sensitizers according to the OECD guideline criteria.

**Figure S2** shows the cell viability in response to the individual PAHs. The dashed line represents the IC70 criterion of the OECD guideline.

**2.2. Mixtures**

**Table S2**: IC70 of the individual PAHs in the mixtures.

|  | **Mixtures** | **Acy [µM]** | **An [µM]** | **B[a]A [µM]** | **B[a]P [µM]** | **B[b]F [µM]** | **B[e]P [µM]** | **B[g,h,i]P [µM]** | **B[k]F [µM]** | **Chr [µM]** | **Fla [µM]** | **Fle [µM]** | **Nap [µM]** | **Phe [µM]** | **Pyr [µM]** | **Trp [µM]** |
| --- | --- | --- | --- | --- | --- | --- | --- | --- | --- | --- | --- | --- | --- | --- | --- | --- |
| 1 | B[a]P + Fle + Nap |  |  |  | 13 |  |  |  |  |  |  | 313 | 78 |  |  |  |
| 2 | B[a]P + An + Phe |  | 101 |  | 8 |  |  |  |  |  |  |  |  | 101 |  |  |
| 3 | B[a]P + B[b]F + B[k]F |  |  |  | 3 | 5 |  |  | 3 |  |  |  |  |  |  |  |
| 4 | B[a]P + B[a]A + Chry |  |  | 12 | 3 |  |  |  |  | 2 |  |  |  |  |  |  |
| 5 | B[a]P + B[e]P + B[g,h,i]P |  |  |  | 14 |  | 31 | 333 |  |  |  |  |  |  |  |  |
| 6 | B[a]P + Acy + Fla | 125 |  |  | 14 |  |  |  |  |  | 63 |  |  |  |  |  |
| 7 | B[a]P + Trp + Pyr |  |  |  | 14 |  |  |  |  |  |  |  |  |  | 63 | 17 |

Chemicals were mixed in a fixed concentration ratio based on their individual ECIR1.5 value or cytotoxicity and diluted in 1: 4 serial dilution three times and a dose response study was carried out. The chemical mixtures and vehicle control were treated in triplicate for each experiment. Three technical replicates were used for all each mixtures in at least two independent experiments.

The color coding visually differentiates the irritant and sensitizing potential of the investigated PAH: irritants (An, Acy); severe eye damaging (Trp); sensitizer - weak (An, Fle), moderate (B[a]A, Nap, Phe), strong (B[a]P, B[b ]F, B[k]F); nonsensitizer (Acy, B[e]P, B[g,h,i]P, Fla, Pyr)

Abbreviations: Acy, Acenaphthylene; An, anthracene; B[a]A, benzo[a]anthracene; B[a]P, benzo[a]pyrene; B[b]F, benzo[b]fluoranthene, benzo[e]pyrene, B[e]P; B[g,h,i]P, benzo[g,h,i]perylene; B[k]F, benzo[k]fluoranthene; Chr, chrysene; Fla, fluoranthene; Fle, fluorene; Nap, naphthalene; Phe, phenanthrene; Pyr, pyrene; Trp, triphenylene

**Table S3**: Sum of the toxic activities (S) of the individual PAHs, both alone and when combined in the mixture

|  | **EC1.5_i_**  **[µM]** |  | **EC1.5_m_**  **[µM]** |  |  |  |  |  |  |
| --- | --- | --- | --- | --- | --- | --- | --- | --- | --- |
| **PAH** |  |  | **Mix1** | **Mix2** | **Mix3** | **Mix4** | **Mix5** | **Mix6** | **Mix7** |
| Acy |  |  |  |  |  |  |  | 1.95 |  |
| An | 157.6 |  |  | 2.9 |  |  |  |  |  |
| B[a]P | 13.6 |  | 0.3 | 0.2 | 0.2 | 0.2 | 0.21 | 0.21 | 0.27 |
| B[a]A | 52.9 |  |  |  |  | 0.8 |  |  |  |
| B[b]F | 0.5 |  |  |  | 0.3 |  |  |  |  |
| B[e]P |  |  |  |  |  |  | 0.49 |  |  |
| B[g,h,i]P |  |  |  |  |  |  | 5.2 |  |  |
| B[k]F | 0.4 |  |  |  | 0.2 |  |  |  |  |
| Chr | 3.9 |  |  |  |  | 0.1 |  |  |  |
| Fla |  |  |  |  |  |  |  | 0.98 |  |
| Nap | 38.3 |  | 1.9 |  |  |  |  |  |  |
| Fle | 177.9 |  | 7.8 |  |  |  |  |  |  |
| Phe | 83.4 |  |  | 2.9 |  |  |  |  |  |
| Pyr |  |  |  |  |  |  |  |  | 1.24 |
| Trp |  |  |  |  |  |  |  |  | 0.33 |
| **S** |  |  | **0.09** | **0.07** | **1.20** | **0.06** | **0.02** | **0.02** | **0.02** |

Abbreviations: Acy, Acenaphthylene; An, anthracene; B[a]A, benzo[a]anthracene; B[a]P, benzo[a]pyrene; B[b]F, benzo[b]fluoranthene, benzo[e]pyrene, B[e]P; B[g,h,i]P, benzo[g,h,i]perylene; B[k]F, benzo[k]fluoranthene; Chr, chrysene; Fla, fluoranthene; Fle, fluorene; Nap, naphthalene; Phe, phenanthrene; Pyr, pyrene; Trp, triphenylene


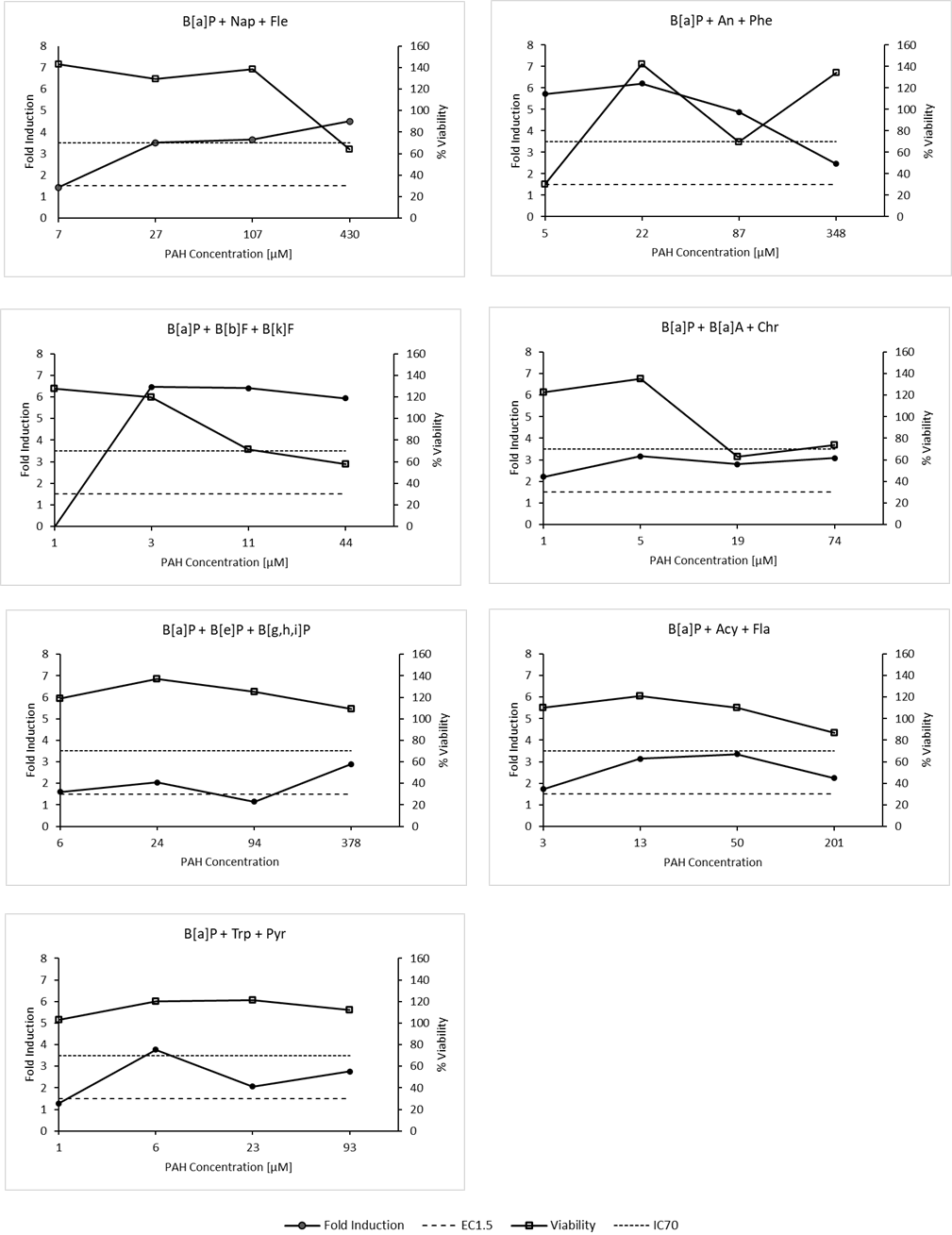


**Fig. S3**: KeratinoSens data of the fold induction and the cytotoxicity of the mixtures.

The graphs show the induction of the luciferase activities (filled circles) and MTT data (open squares) used to determine the cytotoxicity of the mixtures. The dashed line indicates the threshold for significant fold induction, above which mixtures are considered sensitizers. Similarly, the dotted line represents the cytotoxicity threshold.

Abbreviations: Acy, Acenaphthylene; An, anthracene; B[a]A, benzo[a]anthracene; B[a]P, benzo[a]pyrene; B[b]F, benzo[b]fluoranthene, benzo[e]pyrene, B[e]P; B[g,h,i]P, benzo[g,h,i]perylene; B[k]F, benzo[k]fluoranthene; Chr, chrysene; Fla, fluoranthene; Fle, fluorene; Nap, naphthalene; Phe, phenthrene; Pyr, pyrene; Trp, triphenylene
